# CO_2_ Separation with Polymer/Aniline Composite Membranes

**DOI:** 10.3390/polym12061363

**Published:** 2020-06-17

**Authors:** Hwa Jin Lee, Sang Wook Kang

**Affiliations:** 1 Department of Chemistry, Sangmyung University, Seoul 03016, Korea; hwajin96510@naver.com; 2 Department of Chemistry and Energy Engineering, Sangmyung University, Seoul 03016, Korea

**Keywords:** CO_2_, separation, facilitated transport, carrier

## Abstract

Polymer composite membranes containing aniline were prepared for CO_2_/N_2_ separation. Aniline was selected for high separation performance as an additive containing both the benzene ring to interfere with gas transport and an amino group that could induce the accelerated transport of CO_2_ molecules. As a result, when aniline having both a benzene ring and an amino group was incorporated into polymer membranes, the selectivity was largely enhanced by the role of both gas barriers and CO_2_ carriers. Selective layers coated on the polysulfone were identified by scanning electron microscopy (SEM) images and the interaction with aniline in the polymer matrix was confirmed by FT-IR spectroscopy. The binding energy of oxygen in the polymer matrix was investigated by XPS, and the thermal stability of the composite membrane was confirmed by TGA.

## 1. Introduction

Increasing CO_2_ emissions in the environment is leading to global warming, which has become a major concern today [1,2]. Excessive greenhouse gases in the atmosphere cause a variety of environmental problems, such as constant rise in sea level, sea storms, and increased flooding [3,4]. Of the greenhouse gases, CO_2_ is a major cause of global warming, and alone accounts for about 64% for the deterioration of the greenhouse effect [5]. Thus, the efforts to solve these problems and reduce CO_2_ have been researched and reported under various categories such as absorption and adsorption technology [6,7,8].

However, the development of more efficient CO_2_ separation processes has remained only of interest in industrial and academic research, although absorption or adsorption-based processes have been widely used in the industrial field for CO_2_ separation. Especially, membrane-based technology has acquired much attraction recently. It can be utilized in important applications including pure gas supply, natural gas separation (CH_4_/CO_2_), and CO_2_ capture (H_2_/CO_2_ and CO_2_/N_2_) [9,10]. In addition, several advantages, such as easy operation, reliability, environmental friendliness, low cost and energy consumption, are attractive as reducing technology for greenhouse gases. However, for the case of gas separation, gas transport through polymer membranes follows a solution–diffusion mechanism, but the trade-off has been observed between gas permeability and selectivity. Thus, the performance limitation called “Robeson Upper Bound” exists [11]. Due to these limitations [12], facilitated transport membranes have been considered as an effective alternative to overcome this limitation in CO_2_ separation processes [13,14,15]. Facilitated transport is to add a carrier that reacts only with a specific material in an existing medium, so that the transport of a specific material becomes very fast because a carrier-mediated transport is added to the existing fickian transport.

Recently, a growing number of groups has been investigating the facilitated transport applied for CO_2_ separation. Dai et al. reported that polyethylene glycol dimethyl ether (PEGDME, Mn-250 and 500) of different molecular weights was added as a CO_2_-philic additive to Nafion-based membranes [16]. The addition of 40 wt % PEGDME (Mn-250) to the Nafion matrix showed 57.4 barrer of CO_2_ permeability in the dry state. It was 36-fold higher than the original Nafion, and the CO_2_ capture performance was improved. Kline et al. reported on PEO-based crosslinked membranes by systematically varying crosslinking densities and crosslinking heterogeneity [17]. These crosslinked PEO films surpassed the most recent Robeson upper limits for CO_2_/H_2_ and CO_2_/N_2_ separations, making them an attractive membrane for H_2_ purification and carbon capture. Reijerkerk et al. presented the heat and mass transfer properties of a series of mixed membranes prepared with commercially available PEBAX^®^ MH1657 and poly (ethylene glycol) (PEG)-based additives [18]. The additive (PDMS-PEG) was very flexible and permeable, improving the permeability of 530 barrer and CO_2_/H_2_ selectivity at 50 wt% loading. Hanioka et al. reported the SLM (supported liquid membrane) based on a specific ionic liquid to achieve highly-selective and facilitated CO_2_ transport through the membrane [19]. The SLM promoted by the amine-terminated ionic liquid showed high selectivity and high stability for CO_2_ separation from a CO_2_/CH_4_ gas mixture. Zulfiqar et al. reported that polymeric ionic liquids (PILs) served as potential substitutes that could offer a versatile and tunable platform to fabricate a wide range of sorbents for CO_2_ capture particularly for flue gas separation and natural gas purification [20]. On the other hand, Zhang et al. fabricated facilitated transport membranes containing sodium glycine (SG) for enhancing CO_2_ separation performance [21]. The introduced SG provided simultaneously abundant −COO− and −NH_2_ groups as carriers for facilitating CO_2_ transport, and the addition of SG increased the water content in membranes, enhancing CO_2_ solubility. Sun et al. reported that they had succeeded in producing a series of polymers containing a number of secondary amines [22]. These secondary amines provided adequate adsorbate–adsorbent interaction with regard to selective capture of CO_2_. As a result, these materials were reported as producing selective adsorption of CO_2_ and exhibited high CO_2_/N_2_ and CO_2_/CH_4_ selectivity.

Our group has also conducted various studies to increase CO_2_ transport. In research to utilize ionic liquids, composite membranes were prepared containing ZnO nanoparticles and a representative ionic liquid, 1-butyl-3-methylimidazolium tetrafluoroborate (BMIM^+^BF_4_^−^). Consequently, the selectivity and permeance of CO_2_ in the composite membrane were greatly improved to 42.1 and 101 GPU [23]. Furthermore, poly(ethylene oxide) (PEO) composite membranes were prepared for CO_2_/N_2_ separation through the prepared CrO_3_ particles and BMIM^+^BF_4_^−^ dispersion. When compared to the pure PEO membrane and the composite membrane, the permeability increased from 11.0 GPU up to 144 GPU and the selectivity improved from 6.5 to 30. In these researches, CrO_3_ particles increased the solubility of CO_2_, while free imidazolium ions of BMIM^+^BF4^−^ enhanced CO_2_ transport, increasing permeability and selectivity [24]. Furthermore, highly selective composite membranes for CO_2_ were suggested using BMIM^+^BF_4_^−^ and rod-shaped aluminum oxide [25]. As a result, the BMIM^+^BF4^−^/rod shaped Al_2_O_3_ composite obtained a permeance of 39.3 GPU and selectivity of 43.7.

On the other hand, research to utilize both the barrier effect using aromatic rings and the carrier effect was reported [26,27,28]. For example, the impact of 5-hydroxy-isophthalic acid on the facilitated transport of CO_2_ was investigated [26]. When 5-hydroxy-isophthalic acid was incorporated into the poly(ethylene oxide) (PEO) polymer matrix, the membrane separation performance was largely improved, the ideal selectivity 32.4 of CO_2_ to N_2_ and the CO_2_ permeability of 573 barrer were observed. The carboxyl group of 5-hydroxy-isophthalic acid produced a dipole–dipole interaction with the CO_2_ molecule to increase the solubility of the CO_2_ while the benzene rings as barrier effect could reduce N_2_ transport, resulting in high permeability and high selectivity. Furthermore, 1,3,5-benzene tricarboxylic acid was used in polymer composite membranes to achieve improved CO_2_/N_2_ separation performance. Conclusively, the selectivity of CO_2_ increased to 8.5 and CO_2_ gas permeance was 1.2 GPU [27]. For poly(ethylene oxide) (PEO) membranes using 4-hydroxybenzoic acid (4-HBA), the CO_2_ selectivity increased from 1.8 to 23 and CO_2_ permeance was 8.8 GPU [28].

However, a solid state membrane is more desirable for practical application since the liquid state such as ionic liquids showed disadvantages such as penetration into the support, resulting in decreased permeability. In this study, we selected poly(vinyl alcohol) (PVA) containing a hydroxyl group as hydrophilic functional group. It was thought that the PVA as polymer matrix could not easily penetrate into the support when coated on a polysulfone porous support, and the OH groups included could disperse the additives with the effect of enhancing the solubility of CO_2_ molecules. Especially, aniline as an additive was utilized since it was expected that the benzene ring could generate a barrier effect and the amino groups could act as carrier for facilitated transport. Therefore, aniline would help the CO_2_ molecule pass through the membrane.

## 2. Materials and Methods

### 2.1. Materials

Poly(vinyl alcohol) (PVA) (*M*_w_ = 85,000~124,000) and aniline were purchased from Sigma-Aldrich (Saint Quentin Fallavier, France). Distilled water was used as the solvent. The permeance measurement was followed as described previously [29].

### 2.2. Preparation of Membrane

The membranes were prepared using PVA, aniline and distilled water. First, the PVA was added together with distilled water to make a 3 wt % solution. Then aniline was added in various mole ratios. To dissolve evenly the solutes, the solution was stirred one day at 95 °C in an oil bath. The final solution was coated onto polysulfone microporous membrane supports (Toray Chemical Korea Inc., Seoul, Korea) using an RK Control Coater (Model 202, Control Coater RK Print-Coat Instruments Ltd., Litlington, UK). The coated membrane was placed in a vacuum oven to remove the solvents for 3 h.

### 2.3. Permeance Measurements

All gas flow rates refer to gas permeance measurements using a bubble flow meter at room temperature and 2 atmospheres. The unit of gas permeance is GPU and 1 GPU = 1 × 10^−6^ cm^3^ (STP)/(cm^2^ s cmHg).

### 2.4. Characterization

The thickness of the selective layer was investigated using scanning electron microscopy (SEM, JEOL, JSM-5600LV). Fourier transform infrared measurements (FT-IR) were performed on a VERTEX 70 FT-IR spectrometer (BRUKER, Billerica, MA, USA). IR spectra were acquired in the range of the wavenumber from 4000 to 400 cm^−1^, and 16–32 scans were averaged at a resolution of 4 cm^−1^. The weight loss of the composite membrane in flowing N_2_ was confirmed using thermogravimetric analysis (TGA, TGA Q50, TA Instrument, New Castle, DE, USA) at a heating rate of 10 °C/min. X-ray photoelectron spectroscopy (XPS) data were acquired using a PHI 5000 Versa Probe (Ulvac-PHI, Japan) photoelectron spectrometer. This system was equipped with an Al Ka µ-focused monochromator (1486.6 eV) and the detection limit was 0.5 at %. The carbon (C 1s) line at 285.0 eV was used as a reference for determining the binding energies of the O atom.

## 3. Results

### 3.1. Scanning Electron Microscopy (SEM) Images of the Membrane

**Figure 1:**
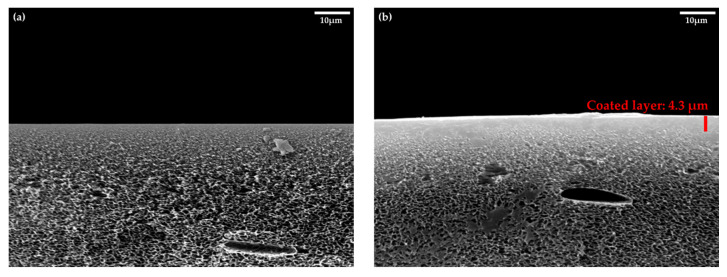
SEM image of (**a**) neat polysulfone and (**b**) 1/0.15 PVA/aniline composite membrane.

The SEM image of polysulfone, a macroporous support, and polysulfone coated with PVA/aniline revealed the presence and thickness of a selective layer of composite membrane. The sponge-like structure of the support was effective for gas permeation (shown in Figure 1a). In addition, as Figure 1b, the PVA/aniline composite membrane had a selective layer, which was a section that contributed to increasing the selectivity of the gas. The average thickness of the selective layer was about 4.3 µm, filling the pores of the support and generating facilitated transport of CO_2_.

### 3.2. Thermogravimetric Analysis (TGA)

**Figure 2:**
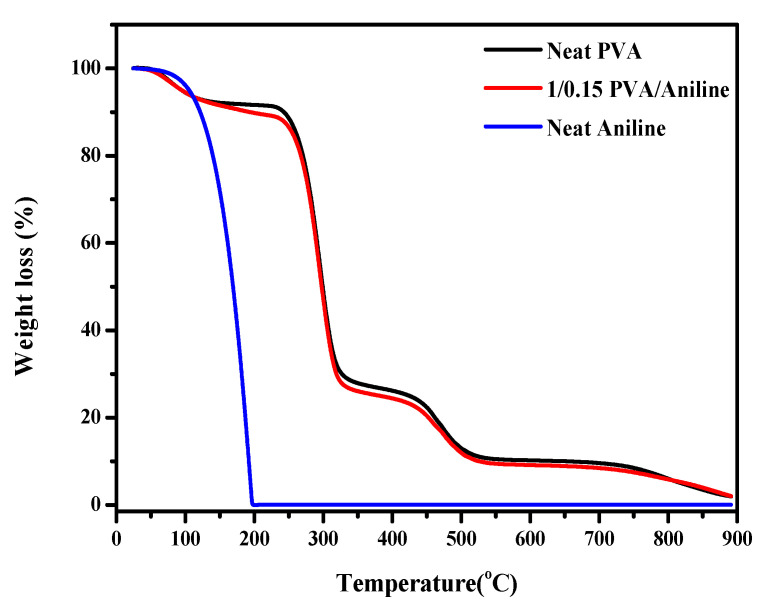
TGA graph of neat PVA, neat aniline and 1/0.15 PVA/aniline composite membranes.

The thermal properties of neat PVA, neat aniline, 1:0.15 PVA/aniline composite membranes were measured through TGA as shown in Figure 2. Figure 2 exhibites the multiple-steps of degradation. Evaporation of the solvent and partial aniline amount occurred from room temperature to about 100 °C. The change of curve in the next step to 235 °C was generated by the melting point of the PVA molecule, and the decomposition around 430 °C was the stage at which the hydroxy group decomposed from the PVA chains. Finally, degradation of the polymer backbone was observed after about 700 °C. When aniline was added to the polymer, the intermolecular force of PVA was reduced by the aniline, and chain-packing was prevented compared to the pure polymer. These steric effects increased the free volume and decreased the thermal stability. These increased free volumes were expected to enhance the gas permeability through increased diffusivity.

### 3.3. X-ray Photoelectron Spectroscopy (XPS)

**Figure 3:**
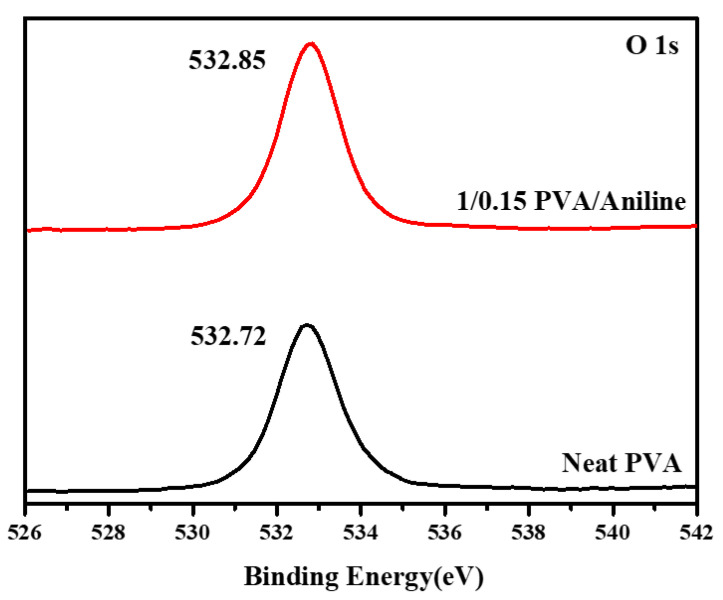
XPS spectra showing binding energy of oxygen in neat PVA and 1/0.15 PVA/aniline composite membranes.

The change in the chemical environment of the O atom in the PVA/aniline composite was analyzed by XPS. As shown in Figure 3, an increase of binding energy for the O atom from 532.72 to 532.85 eV was observed. This increase in binding energy was due to the decrease in the electron density of O atoms, suggesting that there was interaction between the H atom of aniline and the O atom of PVA. In C–O–H bonds of the PVA chain, O was partially negatively charged and formed hydrogen-bonds with N–H of aniline. These interactions caused the electron density of O to be diminished, resulting in the increase of the binding energy.

### 3.4. FT-IR

**Figure 4:**
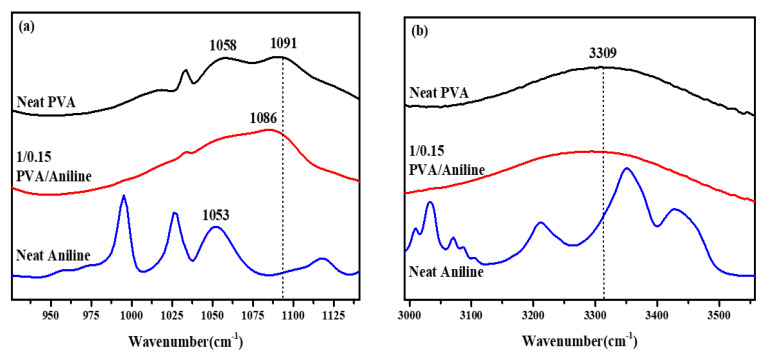
FT-IR spectra of neat PVA and 1/0.15 PVA/aniline composite membranes: (**a**) C–O bond and (**b**) O–H bond.

**Figure 5:**
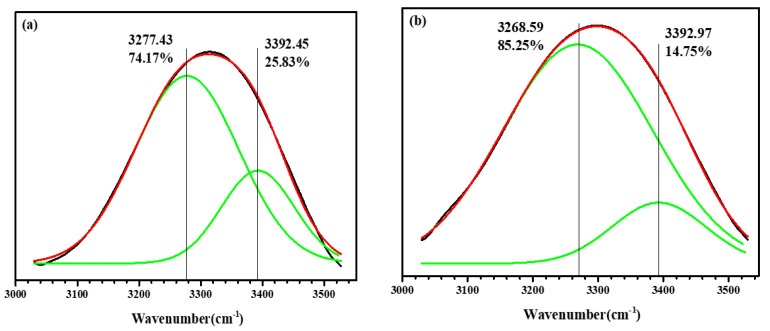
The deconvolution of OH bonding in (**a**) neat PVA and (**b**) 1/0.15 PVA/aniline composite membranes. (red color is original data and green color is the deconvoluted data for each regions)

**Figure 6:**
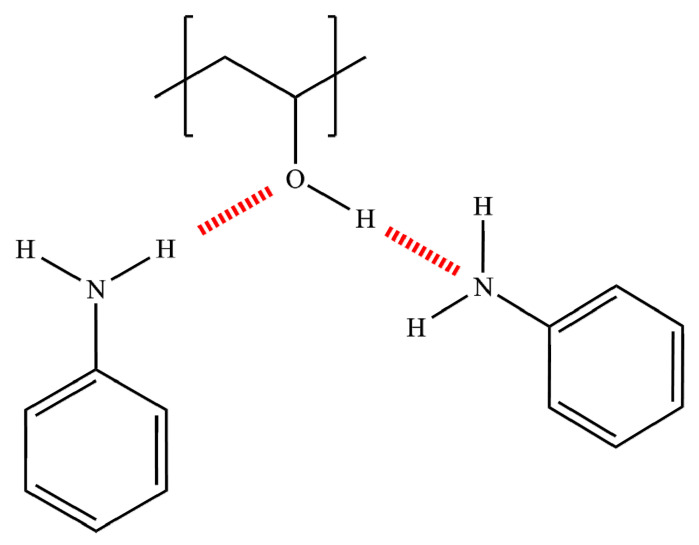
Expected interaction of PVA with aniline.

As shown in Figure 4, to identify the complexation behavior of the functional groups, the FT-IR spectra of neat PVA, neat aniline, and 1:0.15 PVA/aniline composite membrane were measured. Figure 4a shows the peak of the C–O bond in PVA. As shown in Figure 4a, the main peaks of the C–O bond in neat PVA were 1058 and 1091 cm^−1^. The C–O bond of PVA was weakened by the hydrogen bond with aniline, and as a result, the peak was shifted from 1091 to 1086 cm^−1^. Figure 4b shows the IR spectra of the OH bond of PVA, where a change in peak was not observed. The deconvoluted area % for each component is shown in Figure 5 and Table 1. For membranes with aniline embedded in PVA, the area of the left peak increased from 74.17% to 85.25%. This result showed that the strength of the O–H bond was decreased by hydrogen bonding between PVA and aniline. Thus, new interactions between the O atom of PVA and the H atom of aniline, and the H atom of PVA and the N atom of aniline were created as shown in Figure 6, and it was also found that aniline was successfully inserted into the polymer matrix.

### 3.5. Separation Results

**Figure 7:**
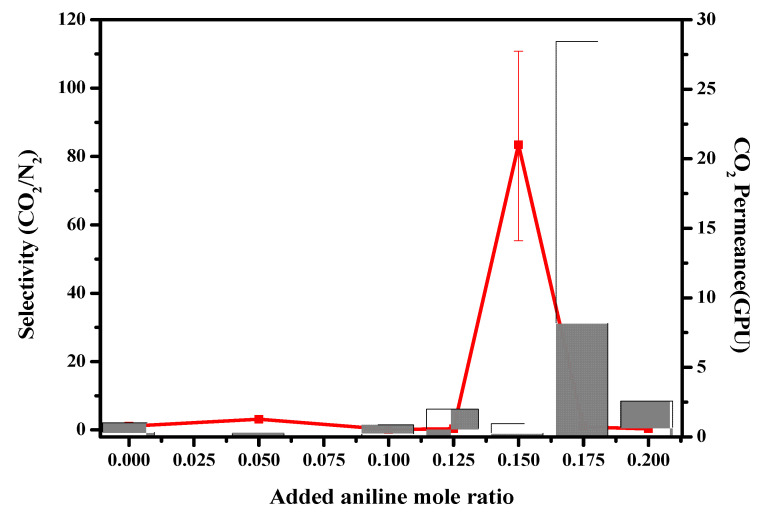
Gas separation performance of PVA/aniline composite membranes. (red color is the selectivity and black square is CO_2_ permeance).

Figure 7 shows the selectivity of CO_2_ according to the mole ratio of aniline added to neat PVA. Neat PVA membrane had almost no selectivity for CO_2_ at 1.1 and permeance was 0.9 GPU. In contrast, the PVA/aniline composite membrane showed the best performance with CO_2_ permeance of 0.8 GPU and selectivity of 83 at 1:0.15 mole ratio of PVA/aniline. These results were obtained through repeated experiments of at least three times, and it was confirmed that the performance was maintained for up to 6 h.

**Scheme 1:**
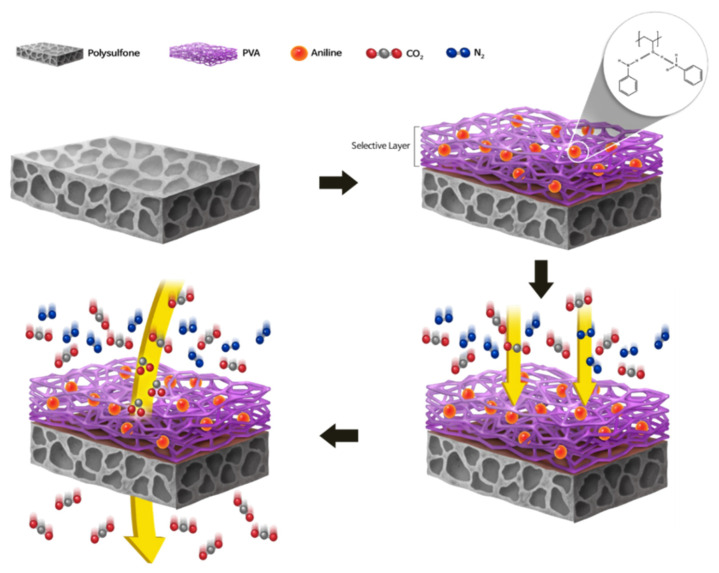
CO_2_ separation for PVA/aniline composite membrane.

This enhanced separation performance meant that aniline had a special role. The first effect was the role as a carrier for facilitated transport. The amino group of the aniline had a basic property that could accelerate CO_2_ transport as a reversible reaction. In particular, CO_2_ molecules, which were originally of linear structure, could give a bent-shape when complexed with the amino group in aniline. The second factor was the barrier effect caused by the benzene ring. The benzene ring had a high electron density, meaning the gas molecules could not be permeated [30]. In addition, aniline could be readily dispersed in the polymer matrix by the interactions with PVA. As a result, facilitation transport occurred in the PVA/aniline composite membrane, as both the solubility and the diffusivity of the CO_2_ molecule increased simultaneously by facilitated transport of the fixed carriers in the solid-state. Data showing the difference between the permeability of the pure polymer and PVA/aniline is not distinguishable, and it seems that aniline acted more as the second factor than the first factor. Scheme 1 shows the separation mechanism of the PVA/aniline composite membrane. After coating PVA/aniline on the porous support of polysulfone, the solubility of CO_2_ increased because aniline in the polymer matrix caused CO_2_ to be largely soluble through the membrane due to facilitated transport. On the other hand, as the transport path of N_2_ increased due to the barrier effect of the benzene ring, the permeability of N_2_ decreased, resulting in the enhancement of selectivity for CO_2_.

However, it was observed that the selectivity decreases after a 0.15 mole ratio. These results were caused by the permeance of all gases enhanced due to the aggregation phenomena of aniline. Above 0.2 mole ratio, a collapsed polysulfone support was observed due to the solvation effect of aniline, resulting in the decrease of gas permeance.

## 4. Conclusions

In this work, we succeeded in providing a highly selective membrane using aniline for facilitated transport for CO_2_ molecules. As a result, the PVA/aniline composite membrane showed a largely enhanced separation performance of about 80 times that of neat PVA membrane with 80 selectivity CO_2_/N_2_ in single gas permeation experiment. These results were due to both the facilitated transport and the barrier effect produced by aniline with enhanced CO_2_ solubility by the OH groups in PVA. The chemical and physical properties of the membranes were characteristics of the membranes which were analyzed by various types of analysis equipment. As a result, it was confirmed that the free volume increase by the additive and hydrogen bonding between the polymer chain and aniline was generated for both the increase of diffusion and CO_2_ solubility with the facilitated transport, resulting in an enhanced separation performance.

## Figures and Tables

**Table 1:** The peak and area % of OH bonding in PVA.

	Peak (cm^−1^)	Area (%)
Neat PVA	3277.43	74.17
1/0.15 PVA/aniline composite membrane	3268.59	85.25
